# Supplementary material for: Quantum simulation of the bosonic Kitaev chain
Source: Nat Commun. 2024 Apr 9;15:3065. doi: 10.1038/s41467-024-47186-8 (PMC11004022; doi:10.1038/s41467-024-47186-8)
Supplement: Supplementary file 1 — Supplementary Information [file 41467_2024_47186_MOESM1_ESM.pdf]

Supplementary Information for Quantum Simulation of the Bosonic Kitaev Chain

Jamal H. Busnaina,[1] Zheng Shi,[1] Alexander McDonald,[2,3] Dmytro Dubyna,[1] Ibrahim Nsanzineza,[1]
Jimmy S.C. Hung,[1] C.W. Sandbo Chang,[1] Aashish A. Clerk,[2] and Christopher M. Wilson[1,*]

[1]*Institute for Quantum Computing and Department of Electrical & Computer Engineering, University of Waterloo, Waterloo, Ontario, N2L 3G1, Canada*
[2]*Pritzker School of Molecular Engineering, University of Chicago, Chicago, IL 60637, USA*
[3]*Institut quantique and Département de Physique, Université de Sherbrooke, Sherbrooke J1K 2R1 QC, Canada*
(Dated: 2024-03-14)

SUPPLEMENTARY NOTE 1 – DEVICE FABRICATION

In the course of fabricating our device, rigorous procedures are undertaken to ensure the cleanliness and precision of the components. The initial step involves the preparation of the wafer surface, wherein a piranha solution, comprising 4 parts of sulphuric acid (H_2SO_4) and 1 part of hydrogen peroxide (H_2O_2), is applied for 15 minutes to eliminate any organic contaminants. The wafer is thoroughly rinsed with deionized water and blow-dried with nitrogen gas. Subsequently, the silicon wafer is soaked in a diluted hydrofluoric acid (2% HF) for 60 seconds to remove the native oxide.

Establishing the circuit layer of our device entails the deposition of a 120 nm-thick aluminum film under vacuum conditions (less than 10^{-8} Torr during the metal deposition) using the Plassys MEB 550 SL3-UHV electron beam evaporator. Following this, a single layer of S1811 resist is spin-coated onto the wafer at 5000 rpm for 60 seconds to achieve $\sim 1.2\mu$m. The next step is optical lithography using a maskless aligner (MLA150 from Heidelberg Instruments). The exposed resist pattern is subsequently developed using MF-319 for 50 seconds. After that the wafer is rinsed with deionized water for 2 min. The exposed aluminum film undergoes wet etching using Transcene's Al Etchant type-A solution. Then, the remaining resist is cleaned with solvents.

For the patterning of Josephson junctions, the JEOL JBX-6300FS 100 kV e-beam lithography system is employed. We have used a bilayer resist of MMA8.5MAA EL13 copolymer as a bottom layer to provide a desired undercut and a top imaging layer of ZEP520A:anisole (1:2). The bottom resist was coated at 3000 rpm for 60 seconds to achieve a thickness of 900 nm. The top imaging resist was coated at 1800 rpm for 60 seconds to achieve 120 nm. After exposing the e-beam patterns, we developed the ZEP520A:anisole (1:2) using N-Amyl acetate (NZED-50) for 2 minutes and a 30-second rinse in isopropanol. We developed the MMA8.5MAA EL13 using a mixture of isopropanol:deionized water (4:1) for 5 minutes followed by a 30-second rinse in isopropanol. The developed trenches are further cleaned using a mixture of isopropanol:ethanol (3:1) followed by a 150-second UV-ozone descumming.

Following e-beam lithography, the sample is introduced into the Plassys MEB 550 SL3-UHV electron beam evaporator. The sample is loaded into the loadlock chamber that is equipped with an ion gun, and the native oxide on aluminum is etched at 400 V and 15 mA for 1 minute to facilitate galvanic contacts. We employ the Dolan-bridge technique, also known as the shadow evaporation technique using a tiltable sample holder stage. We first evaporated 50 nm of aluminum at a rate of 0.2 nm/sec with the sample tilted at +26 degrees. The aluminum film is then oxidized in the oxidation chamber at 1 Torr for 10 minutes using the static-oxidation method. Next, we evaporated 70 nm of aluminum at a rate of 0.2 nm/s with the sample tilted at -26 degrees. Finally, for lift-off, the sample undergoes an overnight (10–12 hours) immersion in a MicroChem Remover PG, which is an NMP based solvent stripper. The samples are then rinsed in 3 beakers of isopropanol and blow-dried with nitrogen gas. The Josephson junctions were then annealed at 200 °C for 10 minutes on a hot plate. For a micrograph figure of the device refer to Ref. [1].

* chris.wilson@uwaterloo.ca

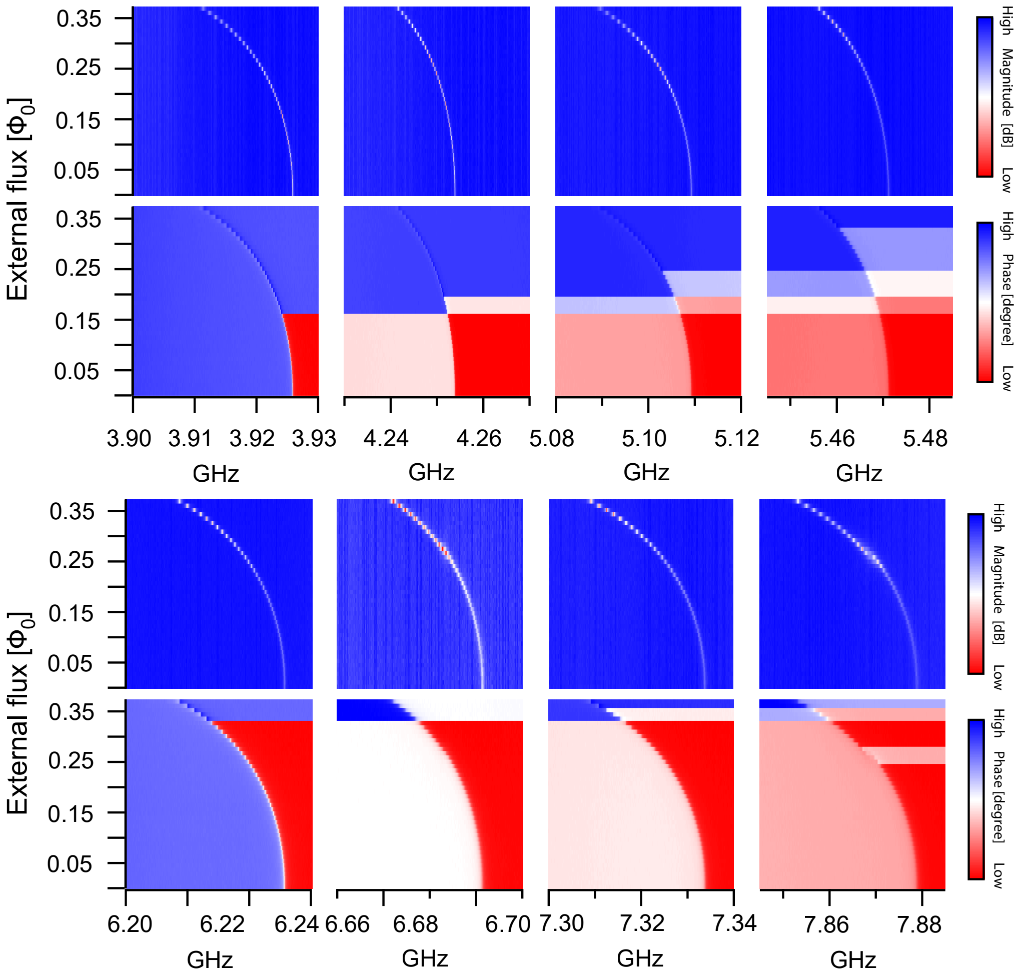

Supplementary Figure 1: Magnitudes and phases of the reflection coefficients as a function of the probe frequency and the external flux. As the external flux increases, the SQUID inductance increases, which then reduces the resonant frequencies of the cavity. As all of the modes are coupled to the SQUID, the above tuning of all eight resonant modes happens simultaneously. The resonant frequencies drop to a minimum when the external flux equals $\Phi_0/2$.

SUPPLEMENTARY NOTE 2 – MEASUREMENT SETUP

In our experimental setup, the superconducting parametric cavity is cooled using a cryogenic-free dilution refrigerator from Bluefors, with a base temperature below 10 mK. The refrigerator comprises five cooling stages, as shown in Supplementary Figure 3. Microwave signals are sent to the device through 50 Ω SMA cables after undergoing significant attenuation in the input line and pump line. The cavity's output is linked to an amplifier chain, starting with a 4 K HEMT amplifier to boost the signal before further amplification at room temperature. A nearby coil is used to tune the DC flux, and the parametric modulation of the SQUID is achieved through the flux pump line connected to an on-chip fast-flux line terminated by a 50 Ω terminator. Circulators are employed between the device and the HEMT. The pump signals are generated using a set of R&S SGS RF sources phase-locked using a 1 GHZ reference. All pump signals are combined at room temperature and fed through the single pump line.

The phase-dependent measurements are performed using Aeroflex instruments. The input signal to the device is generated using an Aeroflex 302X series RF arbitrary wave generator. The fridge output is split into three signals fed to three Aeroflex 303X series RF Digitizers. The S-parameter and spectrum measurements were performed using a Keysight P5024A Vector Network Analyzer.

Our parametric cavity has 13 modes within the measurement bandwidth of 4–12 GHz, which can be readily incorporated into the chain with more flux pumps without frequency crowding. However, the experiments present here utilized eleven microwave sources, reaching the limits of our instrument capacity. The simulation of a 3-mode closed chain demands six microwave sources as pumps. Additionally, two microwave sources are dedicated to upconversion and downconversion

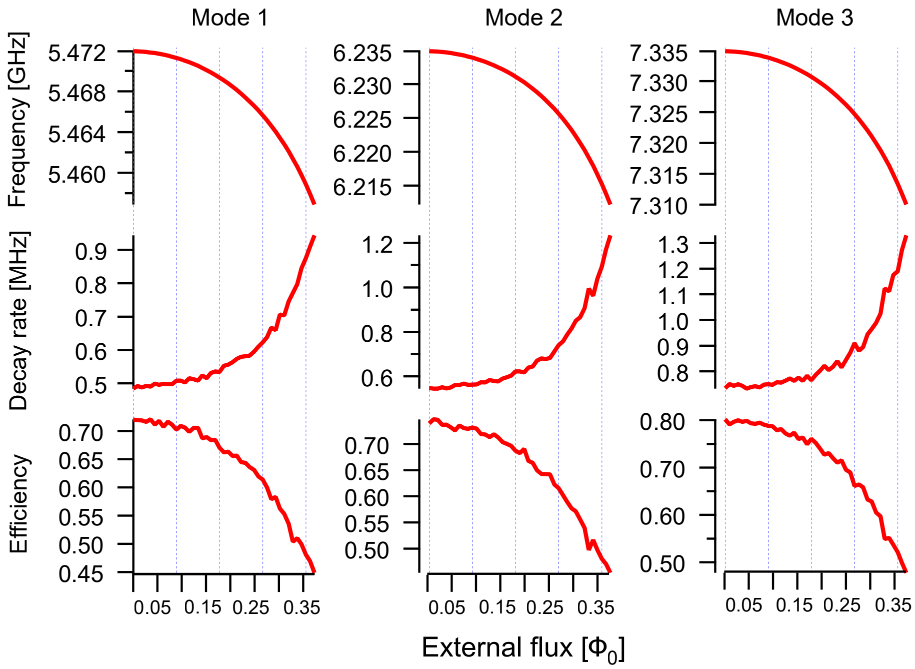

Supplementary Figure 2: Mode parameters as a function of the external flux. We plot the parameters of the three modes chosen to construct the bosonic Kitaev chain.

circuits, while three serve as local oscillators for the RF digitizers used in data acquisition. Calibration of our system is crucial to minimize mode frequency detunings. We note, however, that the resources required for this type of purpose-built quantum simulation circuit is still less than would be required for a gate-based simulation on a universal quantum computer.

As part of future work, we will also explore alternative ways to generate the large number of pumps required to scale the system, for instance using direct-digital synthesis of multiple tones from a single high-speed digital-to-analog converter.

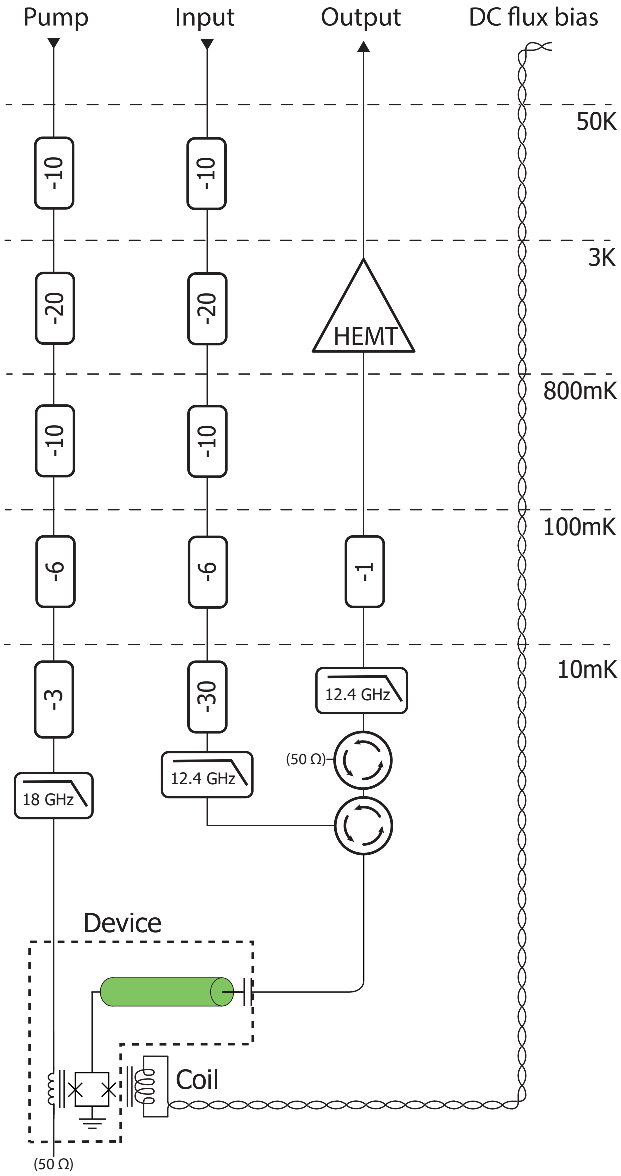

Supplementary Figure 3: Simplified cryogenic microwave network of the experimental setup.

SUPPLEMENTARY NOTE 3 – NONTRIVIAL TOPOLOGY OF THE BOSONIC KITAEV CHAIN

In this section, we discuss the symmetries and the topological invariant of the bosonic Kitaev chain (BKC) model [2], and numerically demonstrate the insensitivity of its non-Hermitian skin effect (NHSE) to symmetry-preserving disorder.

In momentum space, the Hamiltonian of the periodic BKC reads

$$\hat{\mathcal{H}}_{\mathrm{B,p}} = \frac{1}{2}\sum_k \begin{pmatrix} a_k \\ a_{-k}^\dagger \end{pmatrix}^\dagger \mathbf{h}(k) \begin{pmatrix} a_k \\ a_{-k}^\dagger \end{pmatrix}, \tag{1}$$

where the k sum goes over the first Brillouin zone $-\pi < k < \pi$, and the Bloch Hamiltonian has the form

$$\mathbf{h}(k) = \begin{pmatrix} t\cos(\varphi_t - k) & i\Delta\cos k \\ -i\Delta\cos k & t\cos(\varphi_t + k) \end{pmatrix}. \tag{2}$$

The dynamical matrix $\tilde{\mathbf{h}}(k)$ is related to the Bloch Hamiltonian by

$$\tilde{\mathbf{h}}(k) = \sigma_z \mathbf{h}(k) - i\frac{\kappa}{2} = (t\sin\varphi_t \sin k - i\frac{\kappa}{2}) + (t\cos\varphi_t\cos k\sigma_z + i\Delta\cos k\sigma_x), \tag{3}$$

where σ_x, σ_z are Pauli matrices in the Nambu space. The energy spectrum of the periodic chain is composed of the eigenvalues of $\tilde{\mathbf{h}}(k)$,

$$E^{\mathrm{p},\pm}(k) = t\sin\varphi_t\sin k \pm i\sqrt{\Delta^2 - t^2\cos^2\varphi_t}\cos k - i\frac{\kappa}{2}, \tag{4}$$

which becomes Eq. (6) in the main text for a finite-size system. In the topological phase $t|\cos\varphi_t| < \Delta$, as k traverses the first Brillouin zone, the two bands of Eq. (4) trace the same ellipse [Fig. 1(d)] but wind in opposite directions, $E^{\mathrm{p},+}$ clockwise and $E^{\mathrm{p},-}$ counterclockwise. This is because the dynamics of the quadratures of the BKC map to two decoupled Hatano-Nelson chains of opposite chiralities [2].

For any reference point ω in the interior of the spectrum Eq. (4), it is clear that the BKC model has a point gap. However, the winding number with respect to ω identically vanishes as a result of the cancellation between the two bands:

$$W(\omega) = \int_{-\pi}^{\pi} \frac{dk}{2\pi i}\partial_k \ln\det[\tilde{\mathbf{h}}(k) - \omega] = \int_{-\pi}^{\pi}\frac{dk}{2\pi i}\partial_k\Big\{\ln[E^{\mathrm{p},+}(k)-\omega] + \ln[E^{\mathrm{p},-}(k)-\omega]\Big\} = 0. \tag{5}$$

Instead of the full winding number Eq. (5), the topology of the BKC model is characterized by a $\mathbb{Z}_2$ topological invariant, which can be intuitively understood as the winding number of either band in Eq. (4). As we explain below, such a $\mathbb{Z}_2$ invariant arises from a symmetry of our model.

Quite generally, in the Nambu space we have the particle-hole symmetry $\sigma_x\mathbf{h}^T(k)\sigma_x = \mathbf{h}(-k)$. Furthermore, the BKC model in Eq. (2) has no on-site potential and pairing terms. Therefore, a periodic chain with an even number of sites possesses an additional symmetry: under the staggered local gauge transformation $\hat{U}$,

$$\hat{U}\hat{a}_n\hat{U}^\dagger = (-1)^n\hat{a}_n,\ \hat{U}\hat{\mathcal{H}}_{\mathrm{B,p}}\hat{U}^\dagger = -\hat{\mathcal{H}}_{\mathrm{B,p}}. \tag{6}$$

This means the Bloch Hamiltonian satisfies $\mathbf{h}(k) = -\mathbf{h}(k+\pi)$. Making use of the particle-hole symmetry, we find the dynamical matrix $\tilde{\mathbf{h}}(k)$ satisfies a transpose-type symmetry:

$$\sigma_y\tilde{\mathbf{h}}^T(k)\sigma_y = \tilde{\mathbf{h}}(\pi - k). \tag{7}$$

This is a variant of the time-reversal symmetry TRS† discussed in Ref. [3], with $-k$ replaced by $\pi-k$ and the "time-reversal" invariant momenta shifted to $\pm\pi/2$.

One can readily verify that $\tilde{\mathbf{h}}(k)$ has no analogue of the particle-hole symmetry PHS†, sublattice symmetry or pseudo-Hermiticity. Therefore, the BKC model belongs to class AII† in the topological classification of non-Hermitian systems [3]. In the case of a point gap, class AII† possesses a $\mathbb{Z}_2$ topological invariant in the spatial dimension $d = 1$. For the BKC model, such a $\mathbb{Z}_2$ topological invariant $\nu(\omega) \in \{0, 1\}$ can be readily defined on half of the first Brillouin zone [3, 4]:

$$(-1)^{\nu(\omega)} = \mathrm{sgn}\left\{\frac{\mathrm{Pf}[(\tilde{\mathbf{h}}(\frac{\pi}{2}) - \omega)\sigma_y]}{\mathrm{Pf}[(\tilde{\mathbf{h}}(-\frac{\pi}{2}) - \omega)\sigma_y]}\exp\Big\{-\frac{1}{2}\int_{k=-\frac{\pi}{2}}^{k=\frac{\pi}{2}} dk\frac{d}{dk}\ln\det\{[\tilde{\mathbf{h}}(k) - \omega]\sigma_y\}\Big\}\right\}. \tag{8}$$

where for the "time-reversal" invariant momenta $k = \pm\pi/2$, the matrix $[\tilde{\mathbf{h}}(k) - \omega]\sigma_y$ is antisymmetric so that a Pfaffian can be defined. It is straightforward to verify that $\nu(\omega) = 1$ for ω enclosed by the spectrum of the dynamical matrix in the topological phase $t|\cos\varphi_t| < \Delta$, and $\nu(\omega) = 0$ otherwise.

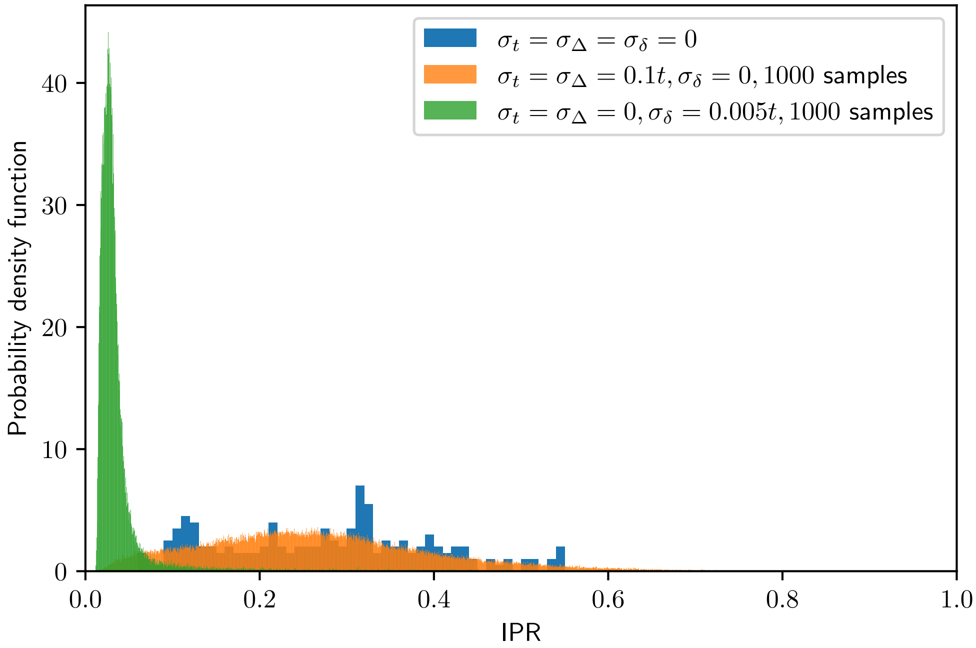

Supplementary Figure 4: Inverse participation ratio (IPR) distributions of a 100-site open chain with different types of disorders. The blue histogram shows the IPR without disorder, the orange one corresponds to moderately strong disorder in both hopping and nearest-neighbor pairing, and the green one corresponds to weak disorder in detuning. A broad distribution indicates the NHSE and thus evidence for topological protection [5].

Robustness against disorder serves as an important indicator of nontrivial topology. Based on the above analysis, we expect our system to be topologically protected against types of disorder that preserve the symmetry Eq. (6), e.g., disorder in hopping t and nearest-neighbor pairing Δ. In fact, this was already analytically demonstrated in Ref. [2] for the special case of $\phi_t = \pi/2$. In contrast, there is no protection against symmetry-breaking disorder, e.g., disorder in detuning $\delta\omega$.

We now present numerical results on the NHSE which support this picture. In class AII† systems, the NHSE is known to originate from non-Hermitian topology [4, 5]. Here we consider the NHSE in an ensemble of disordered open chains. The nearest-neighbor hopping between sites j and $j+1$ is $t_{j,j+1} = t + \sigma_t \eta^t_{j,j+1}$, the nearest-neighbor pairing between sites j and $j+1$ is $\Delta_{j,j+1} = \Delta + \sigma_\Delta \eta^\Delta_{j,j+1}$, and the detuning on site j is $\delta\omega_j = \sigma_\delta \eta^\delta_j$, with $\eta^t_{j,j+1}$, $\eta^\Delta_{j,j+1}$ and η^δ_j independent standard normal random variables. We quantify the NHSE by the inverse participation ratio (IPR) of eigenstate wave functions [6],

$$\mathrm{IPR}^s = \sum_j (|\psi^{\mathrm{p}}_{s,j}|^2 + |\psi^{\mathrm{h}}_{s,j}|^2)^2 / [\sum_j (|\psi^{\mathrm{p}}_{s,j}|^2 + |\psi^{\mathrm{h}}_{s,j}|^2)]^2, \tag{9}$$

where $\psi^{\mathrm{p(h)}}_{s,j}$ is the particle (hole) wave function on site j for eigenstate s. IPR $= 1$ for a completely localized eigenstate, while IPR $= 1/N$ for a completely delocalized eigenstate for a chain with N sites. In the presence of the NHSE, we expect a broad IPR distribution; on the other hand, a sharp IPR distribution whose mean scales as $1/N$ implies the eigenstates are largely delocalized.

In Supplementary Figure 4, the blue histogram shows the IPR distribution of a $\Delta = 0.3t$, $N = 100$ chain with no disorder, normalized to probability densities. The remaining two distributions describe the eigenstate IPRs of 1000 disorder realizations with different types of disorders: the broad orange one has moderately strong disorder in both hopping and nearest-neighbor pairing, $\sigma_t = \sigma_\Delta = 0.1t$, while the narrow green one has a very weak disorder in the detuning, $\sigma_\delta = 0.005t$. One clearly sees that the NHSE persists for hopping and pairing disorder but not for detuning disorder. This is consistent with the picture that our model is topologically protected against types of disorder that preserve the symmetry Eq. (6). We note similar results are also reported by Refs. [4, 5].

SUPPLEMENTARY NOTE 4 – FITTING PROCEDURE

In this section we present the details of fitting the linear model of Eqs. (10) and (11) to the transport data in the open chain and the spectrum data in both open and closed chains. The low-level fitting routines are the SciPy implementations [7] of the limited-memory BFGS algorithm with box constraints (L-BFGS-B) and the Nelder-Mead algorithm [8].

Fitting the transport and spectrum data in the open chain involves three steps:

1. In the first step, we turn off all links and fit the individual spectrum reflection amplitude of each mode as a function of the probe detuning. The fit parameters in this step are the bare cavity mode frequencies $\omega_j^{(0)}$ ($j = a, b, c$), the real and imaginary parts of the complex external coupling rate $\bar{\kappa}_j^{\text{ext}}$, the coupling efficiency $\eta_j = \operatorname{Re} \bar{\kappa}_j^{\text{ext}}/\kappa_j$, a total of $4 \times 3 = 12$ parameters for all three modes (Supplementary Table 1). Here, following Ref. [9], we describe the asymmetry in the resonance lineshape in terms of a phenomenological imaginary part of the external coupling rate $\operatorname{Im} \bar{\kappa}_j^{\text{ext}}$. In practice, instead of manually subtracting the linear backgrounds for the magnitude and phase of the reflection amplitude, we treat the slopes and vertical intercepts of the linear backgrounds as additional fit parameters.

2. In the second step, we activate both hopping terms t_{ab} and t_{bc} but not the pairing terms, and again fit the resulting 3-mode spectrum in a procedure similar to that in Ref. [9], using the individual spectrum fit results from the first step as an initial guess. The fit parameters in this step are $\omega_j^{(0)}$, the real and imaginary parts of $\bar{\kappa}_j^{\text{ext}}$, η_j, t_{ab}, t_{bc}, $\omega_b - \omega_a$ and $\omega_c - \omega_b$, a total of $12 + 4 = 16$ parameters (Supplementary Table 2). Note that the three frequencies ω_j, $j = a, b, c$ are not completely independent because the pairing pumps are not activated. Also, the phases φ_{ab}^t and φ_{bc}^t are both irrelevant as a result of the gauge freedom.

3. In the third step, we activate the pairing terms Δ_{ab} and Δ_{bc} whose strengths have been individually roughly calibrated in advance. Each matrix element of the transport/reflection data has an unknown scale parameter; furthermore, each transport matrix element has an unknown phase offset. We fit all matrix elements simultaneously while fixing $\bar{\kappa}_j^{\text{ext}}$, η_j, t_{ab} and t_{bc} to the values obtained from the second step, assuming they are not strongly affected by the pairing pumps. The fit parameters in this step are the input phase offsets for each input mode j, pump detuning $\delta\omega_j$, Δ_{ab}, Δ_{bc}, φ_{bc}^t, φ_{bc}^Δ, the link phase offsets (e.g., relative to $\varphi_{ab}^+ = 0$) for φ_{ab}^t and φ_{ab}^Δ, the scale factors $C_{jj'}$ for the matrix elements of Eq. (14), and the transported phase offset for each off-diagonal matrix element (S_{ba}, S_{ca}, S_{ab}, S_{cb}, S_{ac} and S_{bc}), a total of $2 \times 3 + 6 + 9 + 6 = 27$ parameters (Supplementary Table 3).

Fitting the spectrum data in the closed chain is similarly a three-step process. The first and the second steps are almost identical to the transport fit, but the second step requires $16 + 2 = 18$ fit parameters (Supplementary Table 4), where the two additional parameters are t_{ca} and one single phase offset corresponding to the gauge-invariant loop phase $\varphi_{ab}^t + \varphi_{bc}^t + \varphi_{ca}^t$. In the third step, we activate the pairing terms, and fit to the resulting spectrum while making the reasonable assumptions that the 2-mode transport contrast (i.e., the relative pairing strength Δ/t) is identical for all three links, and that all η_j remain unaffected by the pairing terms. All ω_j are now fully independent, and all three gauge-invariant phases have their independent offsets. The fit parameters are therefore $\bar{\kappa}_j^{\text{ext}}$ (real and imaginary parts), $\omega_j^{(0)}$, ω_j, t_{ab}, t_{bc}, t_{ca}, Δ/t and the offsets for Θ_j, totaling 19 (Supplementary Tables 5, 6).

Mode j	a	b	c
$\omega_j^{(0)}/2\pi$ [GHz]	5.46641	7.32609	6.22695
σ [KHz]	22	26	26
$\mathrm{Re}\,\bar{\kappa}_j^{\mathrm{ext}}/2\pi$ [MHz]	0.372	0.586	0.461
σ [KHz]	20	27	26
$\mathrm{Im}\,\bar{\kappa}_j^{\mathrm{ext}}/2\pi$ [KHz]	11	12	6
σ [KHz]	21	26	23
η_j	0.628	0.700	0.630
σ	0.033	0.028	0.029

Supplementary Table 1: Uncoupled system parameters and their errors σ extracted from spectrum measurements with all links turned off in the open chain. See the text for definitions of the parameters.

Mode j	a	b	c
$\omega_j^{(0)}/2\pi$ [GHz]	5.46658	7.32596	6.22710
σ [KHz]	103	50	102
$\mathrm{Re}\,\bar{\kappa}_j^{\mathrm{ext}}/2\pi$ [MHz]	0.357	0.602	0.432
σ [KHz]	32	37	35
$\mathrm{Im}\,\bar{\kappa}_j^{\mathrm{ext}}/2\pi$ [KHz]	39	−66	7
σ [KHz]	40	40	44
η_j	0.675	0.646	0.636
σ	0.152	0.052	0.114

Coupling j–j'	a–b	b–c
$t_{jj'}/2\pi$ [MHz]	2.027	2.024
σ [KHz]	69	67
$\lvert\omega_j - \omega_{j'}\rvert/2\pi$ [GHz]	1.85975	1.09909
σ [KHz]	56	62

Supplementary Table 2: System parameters and their errors extracted from spectrum measurements with only hopping and no pairing terms in the open chain.

Mode j	a	b	c
$\delta\omega_j/2\pi$ [MHz]	0.103	0.547	0.057
σ [KHz]	7	37	3

Coupling j–j'	a–b	b–c
$\Delta_{jj'}/2\pi$ [MHz]	0.551	0.617
σ [KHz]	13	13

$C_{jj'}$ $[10^{-4}]$	a	b	c
a	5.618 ± 0.066	3.136 ± 0.051	2.408 ± 0.024
b	3.331 ± 0.051	2.216 ± 0.025	2.078 ± 0.069
c	2.424 ± 0.022	1.918 ± 0.054	2.140 ± 0.073

Supplementary Table 3: System parameters, scale factors $C_{jj'}$ and their errors extracted from transport measurements in the open chain in Figs. 3, 4 and 5. Link phases and phase offsets are not shown here and below; see Methods Sec. D.

Mode j	a	b	c
$\omega_j^{(0)}/2\pi$ [GHz]	4.18661	6.13370	7.52182
σ [KHz]	10	10	11
$\mathrm{Re}\,\bar{\kappa}_j^{\mathrm{ext}}/2\pi$ [MHz]	0.9608	1.5934	1.2511
σ [KHz]	6.5	8.1	7.5
$\mathrm{Im}\,\bar{\kappa}_j^{\mathrm{ext}}/2\pi$ [MHz]	−0.3457	−0.4798	0.0703
σ [KHz]	8.0	10.0	9.2
η_j	0.934	0.951	0.783
σ	0.015	0.009	0.008

Coupling j–j'	a–b	b–c	c–a
$t_{jj'}/2\pi$ [MHz]	2.335	2.051	2.392
σ [KHz]	8.0	8.8	11.2
$\lvert\omega_j - \omega_{j'}\rvert/2\pi$ [GHz]	1.94723	1.38755	3.33478
σ [KHz]	9.5	*	10.6

Supplementary Table 4: System parameters and their errors extracted from spectrum measurements with only hopping and no pairing terms in the closed chain. The entry marked with an asterisk is not an independent fit parameter.

Mode j	a	b	c
$\omega_j^{(0)}/2\pi$ [GHz]	4.18648	6.13363	7.52178
σ [KHz]	4.2	3.9	4.4
$\omega_j/2\pi$ [GHz]	4.18606	6.13333	7.52088
σ [KHz]	4.0	5.5	5.6
$\mathrm{Re}\,\bar{\kappa}_j^{\mathrm{ext}}/2\pi$ [MHz]	0.9388	1.5852	1.2192
σ [KHz]	2.9	3.7	3.3
$\mathrm{Im}\,\bar{\kappa}_j^{\mathrm{ext}}/2\pi$ [MHz]	−0.3529	−0.4523	0.1278
σ [KHz]	3.3	4.1	3.9

Coupling j–j'	a–b	b–c	c–a
$t_{jj'}/2\pi$ [MHz]	2.361	2.103	2.362
σ [KHz]	3.4	3.7	4.1
Δ/t	0.2502		
σ	0.0017		

Supplementary Table 5: System parameters and their errors extracted from spectrum measurements in the closed chain in Fig. 6(a)–(c).

Mode j	a	b	c
$\omega_j^{(0)}/2\pi$ [GHz]	4.18626	6.13357	7.52199
σ [KHz]	11.0	9.7	9.7
$\omega_j/2\pi$ [GHz]	4.18596	6.13332	7.52089
σ [KHz]	8.2	12.2	12.3
$\mathrm{Re}\,\bar{\kappa}_j^{\mathrm{ext}}/2\pi$ [MHz]	0.9016	1.5902	1.2173
σ [KHz]	6.9	8.3	7.0
$\mathrm{Im}\,\bar{\kappa}_j^{\mathrm{ext}}/2\pi$ [MHz]	−0.4290	−0.4800	0.1883
σ [KHz]	6.6	8.8	7.9

Coupling j–j'	a–b	b–c	c–a
$t_{jj'}/2\pi$ [MHz]	2.496	1.994	2.224
σ [KHz]	8.4	11.8	12.6
Δ/t	0.4169		
σ	0.0066		

Supplementary Table 6: System parameters and their errors extracted from spectrum measurements in the closed chain in Fig. 6(d).

[1] C. S. Chang, C. Sabín, P. Forn-Díaz, F. Quijandría, A. Vadiraj, I. Nsanzineza, G. Johansson, and C. Wilson, Physical Review X **10**, 011011 (2020).
[2] A. McDonald, T. Pereg-Barnea, and A. Clerk, Physical Review X **8**, 041031 (2018).
[3] K. Kawabata, K. Shiozaki, M. Ueda, and M. Sato, Physical Review X **9**, 041015 (2019).
[4] N. Okuma, K. Kawabata, K. Shiozaki, and M. Sato, Physical Review Letters **124**, 086801 (2020).
[5] N. Okuma and M. Sato, Physical Review B **103**, 085428 (2021).
[6] G.-F. Guo, X.-X. Bao, H.-J. Zhu, X.-M. Zhao, L. Zhuang, L. Tan, and W.-M. Liu, Communications Physics **6**, 363 (2023).
[7] P. Virtanen, R. Gommers, T. E. Oliphant, M. Haberland, T. Reddy, D. Cournapeau, E. Burovski, P. Peterson, W. Weckesser, J. Bright, *et al.*, Nature methods **17**, 261 (2020).
[8] J. Nocedal and S. Wright, *Numerical Optimization*, Springer Series in Operations Research and Financial Engineering (Springer New York, 2006).
[9] J. S. Hung, J. Busnaina, C. S. Chang, A. Vadiraj, I. Nsanzineza, E. Solano, H. Alaeian, E. Rico, and C. Wilson, Physical Review Letters **127**, 100503 (2021).
